# UTILIZING A LENS OF DIVERSITY, EQUITY, DECOLONIZATION, AND INTERSECTIONALITY IN GERONTOLOGY EDUCATION

**DOI:** 10.1093/geroni/igae098.0305

**Published:** 2024-12-31

**Authors:** Yeonjung Jane Lee

**Affiliations:** University of Hawaiʻi at Manoa, Honolulu, Hawaii, United States

## Abstract

Educational transformation can be catalyzed by engaging in critical self-reflection. In light of the COVID-19 pandemic and deeply ingrained histories of racism and ageism, educators must reflect on their roles and encourage students to critically evaluate the impact of their identities on how they serve older adults. With the aging population becoming more diverse, it is imperative for students to be equipped to work with diverse older adults who come from various cultural backgrounds and life experiences. This session will delve into the presenter’s teaching philosophy and experiences gleaned from instructing and conducting research at a minority-serving institute. With the aim of nurturing high-quality education to support a diverse student body, this session seeks to inspire meaningful dialogue and impart valuable insights about gerontology education. The presentation will cover the discussion integrating diversity, equity, decolonization, and intersectionality perspectives into gerontology education.

